# Clinical and laboratory determinants of treatment response and rifampicin resistance among Xpert MTB/RIF-confirmed extrapulmonary tuberculosis patients: A prospective study from Central India

**DOI:** 10.1371/journal.pone.0361115

**Published:** 2026-11-02

**Authors:** Gautam Bhagwat, Mala Kanthali, Swati Chauhan, Arti Julka, Dharampal Singh Chauhan, Manju Purohit

**Affiliations:** 1 Department of Pathology, R D Gardi Medical College, Ujjain, India; 2 Department of Pathology, Narayan Medical College and Hospital, Bihar, India; 3 Department of Pulmonary Medicine, R D Gardi Medical College, Ujjain, India; 4 Department of Community Medicine, R D Gardi Medical College, Ujjain, India; 5 Central Research Laboratory, R D Gardi Medical College, Ujjain, India; 6 Department of Global Public Health, Karolinska Institutet, Stockholm, Sweden; Hangzhou Red Cross Hospital, CHINA

## Abstract

Extrapulmonary tuberculosis (EPTB) contributes significantly to the global tuberculosis burden, yet treatment response and drug resistance remain poorly characterized. India, a high- tuberculosis burden country, faces growing challenges of delayed diagnosis, treatment and drug-resistant tuberculosis, particularly in extrapulmonary forms. This study evaluated early clinical response to therapy and identified factors associated with rifampicin resistance among patients with EPTB. A prospective observational study was conducted at a tertiary care centre in central India between 2018 and 2024. A total of 532 patients with microbiologically confirmed EPTB, based on the Xpert MTB/RIF assay, were included. The study collected baseline demographic, clinical, and laboratory data. Clinical response at 8 weeks was assessed using standardized criteria. Multivariable logistic regression analysis identified factors associated with slow clinical response and rifampicin resistance. A total of 5,764 presumptive EPTB samples were tested using the Xpert MTB/RIF assay. Among these, 532 patients had confirmed EPTB. Rifampicin resistance was detected in 36 patients (6.7%), while 496 patients had rifampicin-sensitive disease and were included in the response analysis. Among these patients, 113 (22.8%) demonstrated slow clinical response. Slow response showed independent association with HIV co-infection, diabetes mellitus, malnutrition, chronic obstructive pulmonary disease, prolonged symptom duration, and non-lymph node disease, particularly abdominal and genitourinary tuberculosis. Socioeconomic factors, such as rural residence and lower educational status, also showed independent association. Elevated inflammatory markers, including erythrocyte sedimentation rate and C-reactive protein, showed independent association with slow clinical response. Rifampicin resistance showed independent association with previous tuberculosis, HIV infection, male sex, and disseminated disease. A significant overlap existed between delayed clinical response and drug resistance, as rifampicin resistance occurred more frequently among slow responders than among responders (12.3% vs. 4.1%, *p* = 0.002). Nearly one-quarter of patients with EPTB demonstrated delayed clinical response at 8 weeks. Both host-related and disease-related factors showed association with treatment response, while rifampicin resistance showed strong association with prior tuberculosis and immunosuppression. The overlap between slow clinical response and drug resistance highlighted the importance of early molecular diagnostics and targeted clinical monitoring in high-risk patients.

## Introduction

Tuberculosis remained a major global public health challenge, with an estimated 10.6–10.7 million incident cases and approximately 1.3 million deaths annually, and it disproportionately affected low- and middle-income countries [1]. While pulmonary tuberculosis accounted for the majority of cases, extrapulmonary tuberculosis (EPTB) represented a substantial and increasingly recognized component of the disease burden, and it comprised approximately 20% of all tuberculosis cases and up to 40% among immunocompromised individuals and children [2,3]. EPTB involved virtually any organ system, such as lymph nodes, pleura, central nervous system, bones, genitourinary tract, and abdomen, and this resulted in highly heterogeneous clinical presentations [2,4,5].

The diagnosis and management of EPTB remained challenging. Compared with pulmonary tuberculosis, EPTB was often paucibacillary, presented with non-specific symptoms, and frequently required invasive procedures for microbiological confirmation [2,6]. These challenges contributed to delayed diagnosis and treatment initiation, and this delay adversely affected clinical outcomes [7]. In addition, variability in disease site, bacillary burden, host immune response, and drug penetration across anatomical compartments contributed to heterogeneity in treatment response [4]. Despite standardized treatment regimens under national programmes, a subset of patients demonstrated delayed or suboptimal clinical improvement and required prolonged therapy or closer monitoring.

While associated factors of treatment outcomes were extensively studied in pulmonary tuberculosis, evidence specific to EPTB remained limited and inconsistent [4]. Existing studies suggested that factors such as diabetes mellitus, HIV co-infection, undernutrition, and extensive disease influenced treatment outcomes; however, most available data were retrospective, heterogeneous, or focused on long-term outcomes rather than early treatment response [4,5,8]. Early clinical response, particularly within the initial 8 weeks of therapy, provided a critical window for identification of patients at risk of poor outcomes and allowed timely intervention and individualized management.

In parallel, the emergence of drug-resistant tuberculosis, including rifampicin-resistant disease, posed a significant threat to tuberculosis control efforts. Although the burden and determinants of drug resistance were well characterized in pulmonary tuberculosis, data in EPTB remained sparse. In addition, the relationship between delayed clinical response and underlying drug resistance in EPTB remained poorly understood. Identification of overlapping clinical and laboratory factors of slow response and drug resistance could facilitate early risk stratification and guide targeted diagnostic and therapeutic approaches.

Given these gaps, especially in endemic regions such as Central India, a need existed for prospective studies that evaluated early treatment response and its determinants in microbiologically confirmed EPTB. In this context, the present study aimed to identify baseline clinical, demographic, and laboratory predictors of slow clinical response (defined at 8 weeks of standard therapy) among EPTB patients and also determine the proportion and associated factors in rifampicin-resistant EPTB identified at diagnosis from this population.

## Methodology

### Study design and setting

This prospective observational study was conducted at Chandrikaben Rashmikant Gardi Hospital (CRGH), a tertiary care teaching hospital in Ujjain, Madhya Pradesh, India. The hospital serves a mixed urban and rural population and functions as a referral centre for tuberculosis, including complex extrapulmonary cases. The institution is integrated into the National Tuberculosis Elimination Programme (NTEP), and it follows standardized diagnostic and treatment protocols as per national guidelines. The study period extended from 1 June 2018–29 December 2024.

### Study population

All consecutive patients of any age and sex with presumptive EPTB were screened. Patients were included if they had microbiologically confirmed EPTB based on detection of *Mycobacterium tuberculosis* in extrapulmonary specimens using the Xpert MTB/RIF assay. Patients were excluded if they had isolated pulmonary tuberculosis, prior anti-tuberculosis treatment that exceeded two months before enrolment, critical illness that precluded clinical assessment, or refusal to provide informed consent. Patients underwent clinical evaluation for concurrent pulmonary tuberculosis, which included chest radiography where feasible; however, individuals with microbiologically confirmed extrapulmonary disease were retained even when pulmonary involvement could not be completely excluded. Patients aged <14 years were categorized as paediatric, and those ≥14 years were categorized as adults.

### Data collection procedures

All eligible patients underwent prospective enrolment, and trained research staff collected data using structured case record forms. The study recorded sociodemographic characteristics, clinical features, comorbidities (including HIV infection, diabetes mellitus, and chronic obstructive pulmonary disease), prior tuberculosis history, substance use, site of disease, and relevant laboratory and radiological findings. Microbiological data included Xpert MTB/RIF results (including rifampicin resistance), smear microscopy where available, mycobacterial culture, and line probe assay for drug-resistant cases. Diagnosis and management followed WHO and NTEP guidelines.

### Follow-up and outcome assessment

Patients were followed at 15 days, 4 weeks, 8 weeks, and 6 months, and thereafter monthly until completion of the continuation phase (or longer if required). Given the limited utility of microbiological endpoints in EPTB, treatment response was assessed using a standardized composite clinical framework at each visit as per programmatic design [9].

The parameters were systematically evaluated and compared with baseline values: 1. Symptom assessment: Key symptoms (fever, pain, swelling, discharge, or site-specific complaints) were recorded and graded by the patient report using a semi-quantitative scale (0–100%), categorized as 25%, 50%, 75%, or 100% improvement relative to baseline. 2. Objective clinical findings: Physical examination included measurement of lesion size (e.g., lymph node diameter using a measuring tape), presence of tenderness, sinus, or discharge. A reduction in lesion size was quantified, with ≥50% reduction considered significant improvement. 3. Nutritional status (weight change): Body weight was measured at each visit. Percentage weight change from baseline was calculated, with >5% gain considered clinically meaningful. 4. Radiological and/or laboratory parameters (where applicable): Imaging (ultrasound, CT, MRI) or laboratory markers (e.g., erythrocyte sedimentation rate, C- reactive protein) were assessed in selected cases based on disease site and clinical indication, and categorized as improved, unchanged, or worsened. To ensure consistency, all assessments were performed using a standardized follow-up proforma, with direct comparison to baseline findings. The primary endpoint was determination of early clinical response at 8 weeks. The secondary endpoint was identification of rifampicin resistance and its predictors. The final study endpoint was overall response to treatment. Patients with incomplete follow-up or missing key data were excluded from analysis.

### Definitions

Patients were categorized at 8 weeks, corresponding to the end of the intensive phase of first-line anti-tubercular therapy under national programmatic guidelines. Early clinical responders were defined as patients fulfilling at least three of the following criteria: [1] ≥50% improvement in symptom score based on patient-reported semi-quantitative assessment; [2] ≥50% reduction in objective lesion size or site-specific findings; [3] ≥5% weight gain from baseline and [4] improvement in EQ-VAS score or radiological and laboratory parameters, where available, as described previously [9,10].

Patients not fulfilling responder criteria at 8 weeks were categorized as slow clinical responders. Additional definitions included underweight (BMI < 18.5 kg/m²), anaemia (haemoglobin <10 g/dL), elevated erythrocyte sedimentation rate (>50 mm/hr), elevated C-reactive protein (>10 mg/L), prolonged symptom duration (>12 weeks prior to diagnosis), disseminated disease (≥2 non-contiguous sites), and rifampicin resistance (Xpert MTB/RIF and/or confirmatory testing).

### Statistical analysis

Statistical analysis was performed using SPSS version 29 (IBM Corp., NY, USA). Continuous variables were summarized using median and interquartile range (IQR), and categorical variables as frequencies and percentages. Group comparisons used the chi-square or Fisher’s exact test for categorical variables and Student’s t-test. A p-value <0.05 indicated statistical significance. Logistic regression analysis examined factors associated with slow clinical response and rifampicin resistance. The study selected variables for multivariable analysis based on statistical significance in univariate analysis (*p* < 0.05) and clinical relevance, with attention to prevention of overfitting. The analysis restricted the number of variables in accordance with the events-per-variable principle. Adjusted odds ratios (aORs) with 95% confidence intervals (CIs) were reported. Model diagnostics included assessment of multicollinearity using variance inflation factors (<2.0) and evaluation of model fit using the Hosmer–Lemeshow test (*p*- > 0.05). Exploratory Kaplan–Meier analysis was additionally performed to assess time to clinical improvement according to rifampicin resistance status. Missing data remained minimal and were handled using complete-case analysis.

### Ethical considerations

The study protocol was reviewed and approved by the Institutional Ethics Committee of Ruxmaniben Deepchand Gardi Hospital (Approval No: IEC 10/2018 A). Written informed consent was obtained from all participants before enrolment. For illiterate patients, consent was taken in the presence of a witness and in the case of paediatric patients from guardians or parents. Patient confidentiality was strictly maintained, with identifiers removed during analysis.

## Results

### Study population and rifampicin resistance profile

A total of 5,764 presumptive EPTB samples were tested using the Xpert MTB/RIF assay during the study period. Of these, 559 patients were identified as EPTB-positive with valid results. Twenty-seven patients were excluded due to critical illness (n = 4), prior anti-tuberculosis treatment exceeding 2 months (n = 9), or loss to follow-up (n = 14). Thus, 532 eligible patients were included in the analysis, of whom 36 were diagnosed with rifampicin-resistant tuberculosis, while 496 were rifampicin-sensitive (Fig 1). Patients with rifampicin-resistant disease (n = 36) were excluded from the primary 8-week response analysis because they received individualized drug-resistant treatment regimens.

**Fig 1:**
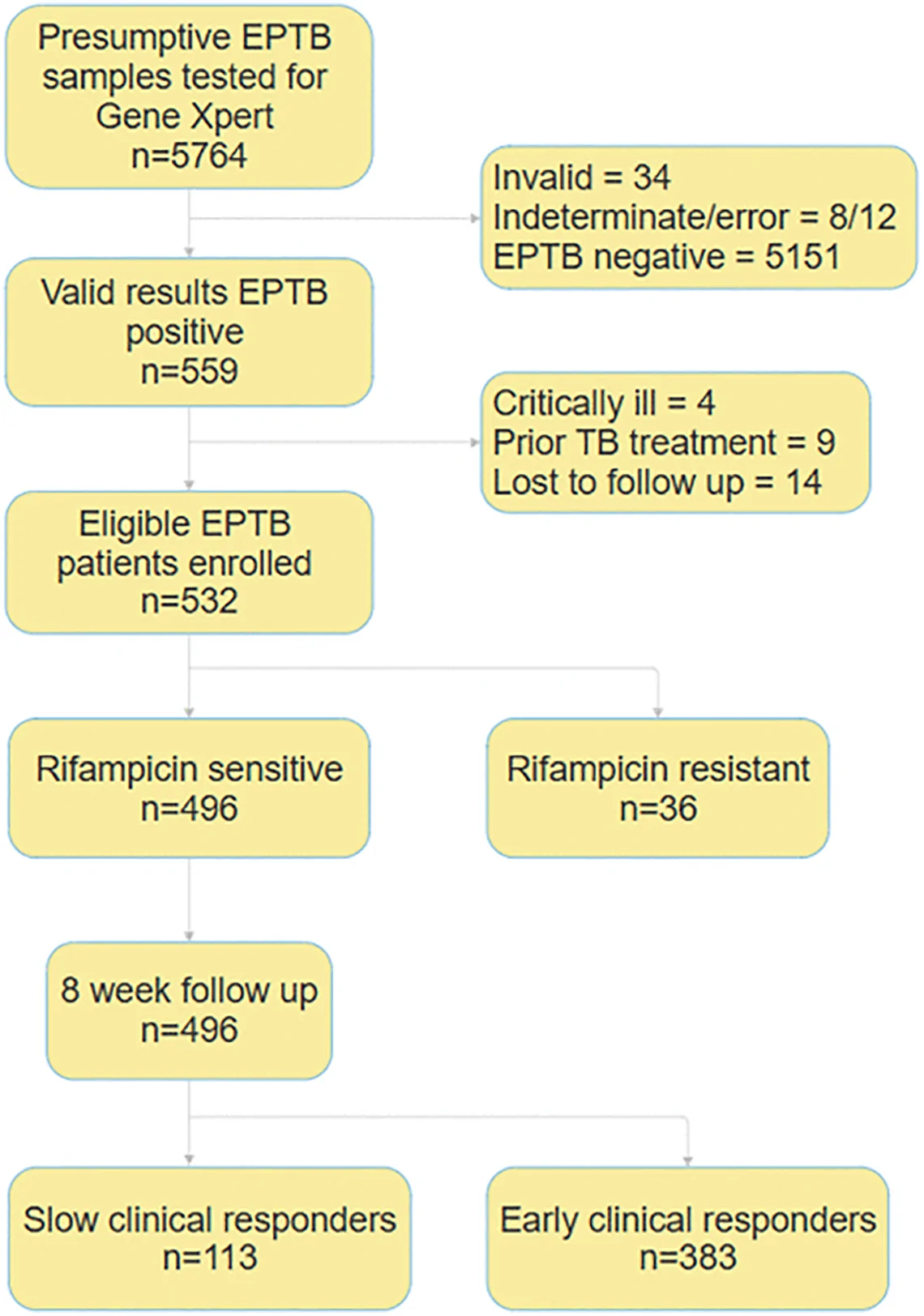
Flow diagram of patient enrolment and analysis.

Patients commonly presented with fever (78%), weight loss (51.3%), and night sweats (28%). The median duration of symptoms prior to diagnosis was 7 weeks (IQR: 5–12 weeks). Laboratory abnormalities included elevated erythrocyte sedimentation rate (>50 mm/hr, 54.8%), anaemia (haemoglobin <10 g/dL, 42.8%), and elevated C-reactive protein (>10 mg/L, 53%). Comorbid conditions included diabetes mellitus, chronic obstructive pulmonary disease, and HIV infection.

As shown in Table 1, rifampicin resistance was significantly more common among younger patients (≤30 years, *p* = 0.006) and males (*p* = 0.009). HIV co-infection (*p* = 0.013), previous history of tuberculosis (*p* < 0.001), and family history of tuberculosis (*p* = 0.017) were also associated with resistance. Lifestyle factors, including smoking and alcohol use, were more frequently observed among rifampicin-resistant patients (*p* = 0.04). Disseminated tuberculosis was significantly associated with rifampicin resistance (*p* = 0.001). Although diabetes and undernutrition were more frequent among resistant cases, these differences were not statistically significant. Markers of systemic inflammation, including elevated erythrocyte sedimentation rate (>50 mm/hr; *p* = 0.004) and C-reactive protein (>10 mg/L; *p* = 0.01), were also higher among rifampicin-resistant patients.

**Table 1:** Demographic, clinical, and laboratory characteristics of extrapulmonary tuberculosis patients (N = 532).

Characteristics n (%)	RIF-Sensitive (n = 496)	RIF-Resistant (n = 36)	*p*-value
**Demographic Characteristics**			
Age ≤ 30 years,	158 (31.9)	20 (55.5)	0.004
Age 31–45 years	167 (33.7)	4 (11.1)	
Age > 45 years	171 (34.5)	12 (33.3)	
Male sex	215 (43.3)	24 (66.7)	0.009
Rural residence	240 (48.4)	21 (58.3)	0.31
**Clinical Risk Factors**			
Diabetes mellitus	66 (13.3)	8 (22.2)	0.18
HIV infection	21 (4.2)	5 (13.9)	0.013
Underweight (BMI < 18.5 kg/m²)	310 (62.5)	25 (69.4)	0.28
Previous TB history	34 (6.9)	12 (33.3)	<0.001
Family history of TB	54 (10.9)	9 [25]	0.017
Smoking/alcohol use	73 (14.7)	10 (27.8)	0.04
**Disease Characteristics**			
Lymph node/abscess TB	285 (57.5)	15 (41.7)	0.13
Pleural TB	107 (21.6)	4 (11.1)	0.23
Cerebral TB	17 (3.4)	3 (8.3)	0.08
Disseminated TB	33 (6.7)	8 (22.2)	0.001
Abdominal/Genitourinary TB	56 (11.3)	3 (8.3)	0.74
**Laboratory Parameters**			
Anaemia (Hb < 10 g/dL)	212 (42.7)	16 (44.4)	0.92
Erythrocyte sedimentation rate >50 mm/hr	262 (52.8)	30 (83.3)	0.004
C-reactive protein >10 mg/L	255 (51.4)	27 (75)	0.01

*TB- Tuberculosis; RIF-Rifampicin; BMI- body mass index

### Clinical response at 8 weeks

Among 496 patients with available follow-up at 8 weeks, 22.8% demonstrated a slow clinical response, while 77.2% were classified as responders. As shown in Table 2, patients with slow clinical response were older (mean age 38.5 ± 14.9 vs 33.9 ± 15.3 years, *p* = 0.003), more likely to reside in rural areas (*p* = 0.001), and had lower educational status (*p* = 0.014). Comorbid conditions were significantly more frequent among slow responders, including HIV co-infection (*p* < 0.001), diabetes mellitus (*p* < 0.001), malnutrition (*p* < 0.001), and chronic obstructive pulmonary disease (*p* = 0.003). In terms of disease characteristics, non-lymph node forms such as abdominal (*p* = 0.001), genitourinary (*p* = 0.003), and central nervous system tuberculosis (*p* = 0.03) were more common among slow responders, whereas lymph node disease was associated with a favourable response (*p* < 0.001). Patients with prolonged symptom duration (≥8 weeks), fever, weight loss, draining sinus, and neurological deficits were significantly associated with slow clinical response. Laboratory abnormalities, including anaemia, hypoalbuminemia, elevated erythrocyte sedimentation rate, and raised C-reactive protein, were also more frequent in this group. A higher bacillary load (Ct ≤ 22) showed a trend towards association (*p* = 0.03). Rifampicin resistance was significantly more common among slow responders (*p* = 0.002).

**Table 2:** Comparison of baseline demographic, clinical, and laboratory characteristics between slow clinical responders and responders at 8 weeks of treatment (n = 496).

Variable	Slow Clinical Responders (n = 113)	Responders (n = 383)	*p*-value
**Demographic**			
Age, years (mean ± SD)	38.5 ± 14.9	33.9 ± 15.3	0.003
Female sex	66 (58.4)	207 (54.0)	0.55
Rural residence	83 (73.5)	160 (41.8)	0.001
Low education (<secondary)	84 (74.3)	229 (59.8)	0.014
**Comorbidities**			
HIV co-infection	17 (15)	8 (2.1)	<0.001
Diabetes mellitus	35 (31)	34 (8.9)	<0.001
Underweight (BMI < 18.5 kg/m²)	54 (47.8)	107 (27.9)	<0.001
Hypertension	9 (8)	22 (5.7)	0.39
Chronic obstructive pulmonary disease	11 (9.7)	10 (2.6)	0.003
Concomitant pulmonary TB	9 (8)	28 (7.3)	0.99
Previous history of TB	14 (12.4)	26 (6.8)	0.07
Family history of TB	18 (15.9)	39 (10.2)	0.09
**Site of EPTB**			
Lymph node/abscess	35 (31)	245 (64.0)	<0.001
Pleural	28 (24.8)	74 (19.3)	0.19
Genitourinary	9 (8)	9 (2.3)	0.003
Abdominal	18 (15.9)	18 (4.7)	0.001
Cerebral	8 (7.1)	10 (2.6)	0.03
**Symptoms**			
Symptom duration ≥8 weeks	70 (61.9)	153 (39.9)	0.001
Fever ≥2 weeks	66 (58.4)	160 (41.8)	0.002
Weight loss >5%	62 (54.9)	147 (38.4)	0.002
Night sweats	36 (31.8)	84 (22.0)	0.03
Localized pain/swelling	34 (30.1)	105 (27.4)	0.59
Draining sinus	21 (18.6)	28 (7.3)	0.001
Neurological deficit	15 (13.2)	9 (2.3)	<0.001
**Laboratory findings**			
Haemoglobin <10 g/dL	57 (50.4)	167 (43.6)	0.19
Serum albumin <3.5 g/dL	41 (36.2)	93 (24.2)	0.01
Erythrocyte sedimentation rate >50 mm/hr	72 (63.7)	170 (44.3)	<0.001
C-reactive protein >10 mg/L	77 (68)	185 (48.3)	<0.001
Leucocytosis (>11 × 10^9^/L)	27 (23.8)	63 (16.4)	0.09
**Microbiological findings**			
EPTB smear positive	13 (11.5)	24 (6.2)	0.07
High bacillary load (Ct ≤ 22)	26 (23)	53 (13.8)	0.03

*TB- Tuberculosis; EPTB-extrapulmonary Tuberculosis

### Factors associated with slow clinical response

On univariate analysis, multiple variables were associated with slow clinical response. In the multivariable logistic regression model (Table 3), rural residence (adjusted OR [aOR] 2.34, 95% CI 1.48–3.71; *p* < 0.001) and low educational status (aOR 1.62, 95% CI 1.03–2.56; *p* = 0.038) remained significant sociodemographic associated factors. Among comorbidities, HIV co-infection, diabetes mellitus, malnutrition and chronic obstructive pulmonary disease were independently associated with slow response. Disease site was an important determinant, with lymph node/abscess tuberculosis being protective, while abdominal and genitourinary tuberculosis were associated with poorer outcomes. Neurological deficit and prolonged symptom duration were also significantly associated. Elevated inflammatory markers, including erythrocyte sedimentation rate (*p* = 0.027) and C-reactive protein (*p* = 0.001), along with anaemia (*p* = 0.046), independently associated with slow clinical response.

**Table 3:** Factors associated with slow clinical response among extrapulmonary tuberculosis patients (N = 496).

Factors	Crude OR (95% CI)	*p*-value	Adjusted OR (95% CI)	*p*-value
Rural residence	3.56 (2.36–5.38)	<0.001	2.34 (1.48–3.71)	<0.001
Low education (<secondary)	1.87 (1.23–2.84)	0.003	1.62 (1.03–2.56)	0.038
HIV co-infection	8.32 (3.62–19.13)	<0.001	5.18 (2.01–13.34)	<0.001
Diabetes mellitus	4.36 (2.65–7.19)	<0.001	3.21 (1.77–5.82)	<0.001
Underweight (BMI < 18.5)	2.28 (1.53–3.41)	<0.001	2.04 (1.26–3.30)	0.004
Chronic obstructive pulmonary disease	4.00 (1.79–8.94)	0.001	2.82 (1.12–7.11)	0.027
Lymph node/abscess TB	0.24 (0.16–0.36)	<0.001	0.32 (0.19–0.53)	<0.001
Genitourinary TB	3.52 (1.47–8.47)	0.004	2.71 (1.01–7.27)	0.047
Abdominal TB	4.04 (2.08–7.84)	<0.001	3.06 (1.42–6.61)	0.004
Cerebral TB	2.89 (1.17–7.14)	0.034	2.36 (0.91–6.08)	0.077
Symptom duration ≥8 weeks	1.86 (1.23–2.81)	0.004	1.84 (1.15–2.94)	0.011
Draining sinus	2.84 (1.55–5.20)	0.001	2.52 (1.18–5.37)	0.017
Neurological deficit	6.04 (2.66–13.69)	<0.001	4.91 (1.85–13.00)	0.001
Haemoglobin <10 g/dL	1.46 (0.97–2.18)	0.087	1.56 (1.01–2.41)	0.046
Erythrocyte sedimentation rate >50 mm/hr	1.62 (1.07–2.46)	0.029	1.68 (1.06–2.65)	0.027
C-reactive protein >10 mg/L	2.26 (1.47–3.46)	<0.001	2.21 (1.40–3.50)	0.001

OR, odds ratio; CI, confidence interval; TB, tuberculosis; CRP, C-reactive protein.

### Factors associated with rifampicin resistance

In the multivariable analysis (Table 4), previous history of tuberculosis was the strongest independent factor associated with rifampicin resistance (*p* = 0.003). HIV co-infection (*p* = 0.027), male sex (*p* = 0.047), and disseminated tuberculosis (*p* = 0.039) were also independently associated with resistance. Younger age (<30 years) showed a borderline association (*p* = 0.056), while elevated C-reactive protein demonstrated a non-significant trend (*p* = 0.08). The multivariable model demonstrated adequate goodness-of-fit (Hosmer–Lemeshow χ² = 2.79, *p* = 0.904). Variance inflation factors ranged from 1.01 to 1.13, indicating no evidence of significant multicollinearity among the predictor variables.

**Table 4:** Multivariate logistic regression model for factors associated with rifampicin resistance (N = 532).

Characteristics	Crude OR (95% CI)	*p-*value	Adjusted OR (95% CI)	*p*-value	VIF
Age < 30 years	2.69 (1.30–5.56)	0.009	2.10 (0.98–4.52)	0.056	1.01
Male sex	2.93 (1.35–6.35)	0.006	2.30 (1.01–5.30)	0.047	1.02
HIV infection	4.22 (1.48–12.07)	0.013	3.50 (1.15–10.60)	0.027	1.08
Previous TB history	6.80 (2.90–15.90)	<0.001	4.20 (1.60–11.00)	0.003	1.09
Disseminated TB	3.84 (1.55–9.51)	0.006	2.90 (1.05–8.00)	0.039	1.13
C-reactive protein >10 mg/L	2.81 (1.24–6.38)	0.017	2.10 (0.90–4.90)	0.080	1.12

OR, odds ratio; CI, confidence interval; TB, tuberculosis; CRP, C-reactive protein. VIF, variance inflation factor.

### Relationship between clinical response, rifampicin resistance, and outcomes

A significant overlap was observed between slow clinical response and rifampicin resistance. A delayed time to clinical improvement was demonstrated among rifampicin-resistant patients (Supplementary S1 Fig). Among slow responders, 12.3% had rifampicin-resistant disease compared to 4.1% among responders (*p* = 0.002). During the six-month follow-up period, nine deaths were recorded. Mortality was higher among patients with slow clinical response (5.3%) compared to responders (*p* = 0.001). Causes of death included disseminated tuberculosis (n = 3), central nervous system tuberculosis (n = 1), HIV-related complications (n = 3), and cardiopulmonary causes (n = 2). Adverse drug reactions were reported in 38 patients (7.1%), most commonly gastrointestinal intolerance, hepatotoxicity, and cutaneous reactions. These were not significantly associated with clinical response.

## Discussion

To the best of current knowledge, this study represents one of the few prospective evaluations of treatment response among microbiologically confirmed EPTB patients from a high-burden setting in central India. Approximately 22.8% of patients demonstrated a slow clinical response at 8 weeks. Slow clinical response was associated with HIV co-infection, diabetes mellitus, abdominal or genitourinary disease, malnutrition, and prolonged diagnostic delay. Rifampicin resistance was identified in 6.7% of patients and showed associations with selected host- and disease-related factors. These findings highlight the multifactorial nature of treatment response in EPTB. In addition, socioeconomic factors such as rural residence and lower educational status were independently associated with slow clinical response, emphasizing the role of healthcare access, health-seeking behaviour, and potential delays in diagnosis and treatment initiation.

The proportion of patients with slow clinical response observed in this study is comparable to prior reports describing delayed improvement or extended treatment duration in EPTB [4,11]. The heterogeneity in response is likely influenced by anatomical site, bacillary burden, host immune status, and variability in drug penetration across disease compartments [2,4]. In the present study, lymph node and abscess-associated disease demonstrated a more favourable clinical course, whereas abdominal, genitourinary, and central nervous system involvement were more frequently associated with slow clinical response. These findings are consistent with the known challenges in managing deeper or less accessible disease sites [4,11]. Prolonged symptom duration prior to diagnosis was an important factor associated with slow clinical response, possibly reflecting more advanced disease at presentation. EPTB often presents with non-specific clinical features and may require invasive procedures for confirmation, contributing to delayed diagnosis and misclassification [11–13]. Given that a proportion of patients with EPTB may have concurrent pulmonary involvement, residual misclassification cannot be entirely excluded. Such delays have been associated with poorer outcomes in previous studies [14], including those evaluating early clinical improvement during tuberculosis treatment. These findings emphasize the need to strengthen early detection pathways, particularly in peripheral and resource-limited settings.

The prevalence of rifampicin resistance (6.7%) observed in this study is consistent with reports from other high-burden settings [15]. Associations with male sex were observed, while younger age showed a trend towards higher odds but did not remain statistically significant after adjustment. Previous studies suggest that younger individuals may reflect transmission-related resistance patterns, although this cannot be confirmed in the present study [16]. Male predominance in drug-resistant tuberculosis has been reported globally and may relate to behavioural and health-seeking differences, although evidence specific to EPTB remains limited [17,18].

Elevated inflammatory markers, including erythrocyte sedimentation rate and C-reactive protein, were independently associated with slow clinical response and showed a positive association with rifampicin resistance. Although non-specific, these markers may reflect underlying disease activity and host inflammatory response. In resource-limited settings, such parameters may assist in identifying patients requiring closer clinical monitoring; however, their role in clinical decision-making requires further validation. Molecular diagnostics, including the Xpert MTB/RIF assay, have improved the detection of EPTB, although performance varies across specimen types and anatomical sites [19,20]. Emerging approaches such as targeted next-generation sequencing offer potential for more comprehensive resistance profiling, but their role in EPTB management requires further evaluation [21–23].

Several factors associated with slow clinical response overlapped with those observed among rifampicin-resistant cases, including HIV co-infection, prior tuberculosis history, disseminated disease, and elevated inflammatory markers. This overlap suggests that delayed clinical improvement and drug resistance may share common underlying factors, such as disease severity and host-related factors. However, the temporal and causal relationship between these variables cannot be established in the present study. Delayed clinical response alone should not be interpreted as a surrogate marker of rifampicin resistance and requires careful clinical and microbiological correlation. While molecular drug susceptibility testing remains essential for confirming drug resistance, patients with delayed clinical response may warrant closer clinical evaluation and, where appropriate, repeat microbiological assessment. Further studies are needed to determine whether integrated clinical and laboratory parameters can improve early identification of drug-resistant EPTB.

Comorbid conditions, particularly diabetes mellitus and HIV infection, were also associated with delayed clinical response. Diabetes has been shown to impair immune function and alter drug pharmacokinetics, which may influence treatment outcomes [24,25]. Similarly, HIV infection modifies host immunity, increases disease dissemination, and is associated with more complex clinical presentations of tuberculosis, including extrapulmonary forms [26–28]. In the present study, HIV co-infection was also more frequently observed among rifampicin-resistant cases, although this finding should be interpreted cautiously due to the limited number of resistance events. Chronic obstructive pulmonary disease was also independently associated with slow clinical response, possibly reflecting compromised systemic and immune function, although evidence in EPTB remains limited. These observations support the importance of integrated clinical management approaches for patients with coexisting conditions, although causal inferences cannot be drawn.

From a programmatic perspective, the findings suggest the potential value of strengthening early diagnosis of EPTB, ensuring access to molecular testing, and providing closer follow-up for patients with identified high-risk characteristics, including comorbid conditions and disseminated disease. By integrating microbiological and clinical parameters, the study contributes to the understanding of heterogeneity in treatment response, an area that remains underexplored in predominantly diagnostic-focused studies. The impact of specific intervention strategies requires further evaluation in prospective and interventional studies.

### Strengths and limitations

This study has several strengths, including its prospective design, systematic follow-up, and relatively large cohort of microbiologically confirmed EPTB patients from a high-burden setting in central India. The use of standardized data collection tools and predefined outcome criteria enhances internal consistency and reduces information bias.

However, several limitations should be acknowledged. First, the composite definition of clinical response, although pragmatic and reflective of routine clinical practice, has not undergone formal validation and may be subject to measurement variability and misclassification. Sensitivity analyses using alternative response thresholds (e.g., ≥ 25% or ≥75%) were not performed, and future studies should validate this outcome against objective clinical, radiological, and microbiological endpoints. Second, although variables for multivariable adjustment were selected based on their clinical relevance and statistically significant univariable associations, the relatively small number of rifampicin-resistant cases (n = 36) may have limited model stability and increased the possibility of overfitting.

Therefore, these findings should be interpreted cautiously and confirmed in larger prospective studies. Some variables, including micronutrient status and pharmacokinetic variability, were beyond the scope of the present analysis and may also influence treatment response. Third, the study evaluated only early (8-week) clinical response without assessing long-term outcomes such as treatment completion, relapse, or post-treatment sequelae. Fourth, as a single-centre study conducted at a tertiary referral hospital, the findings may not be fully generalizable, and reliance on Xpert MTB/RIF limited microbiological assessment to rifampicin resistance.

## Conclusion

Approximately one-quarter of patients with EPTB demonstrated a slow clinical response at 8 weeks. Several demographic, clinical, and laboratory factors were associated with slow clinical response and rifampicin resistance. The observed overlap between delayed clinical response and resistance highlights the need for careful clinical assessment and appropriate use of molecular diagnostics. Further multicentric studies with larger sample sizes and longer follow-up are required to validate these findings and inform clinical and programmatic strategies.

## Supporting information

S1 Fig Kaplan–Meier curves of time to clinical improvement by rifampicin resistance status. Survival curves illustrating differences in time-to-response between rifampicin-resistant and rifampicin-sensitive extrapulmonary tuberculosis patients.
(TIF)

S2 Fig Distribution of erythrocyte sedimentation rate levels among slow clinical responders versus responders. Box-and-whisker plots showing significantly higher baseline erythrocyte sedimentation rate among patients with slow clinical response compared to responders.
(TIF)

S3 Fig Subgroup analysis of patients with slow-clinical response by site of extrapulmonary TB. Bar chart showing proportions of slow response patients across disease sites including lymph node, pleural, Cerebral, abdominal, osteoarticular, and disseminated TB.
(TIF)

S1 Table Baseline demographic and clinical characteristics of the studied extrapulmonary tuberculosis patients (N = 532). (DOCX)

S2 Table Comparison of baseline characteristics according to rifampicin resistance and clinical response status among extrapulmonary tuberculosis patients. (DOCX)
