# Supplementary material for: Clinical and laboratory determinants of treatment response and rifampicin resistance among Xpert MTB/RIF-confirmed extrapulmonary tuberculosis patients: A prospective study from Central India
Source: PLoS One. 2026 Nov 2;21(11):e0361115. doi: 10.1371/journal.pone.0361115 (PMC13726920; doi:10.1371/journal.pone.0361115)
Supplement: Supplementary file 1 [file pone.0361115.s004.docx]

Supplementary Table S1. Baseline demographic and clinical characteristics of the studied extrapulmonary tuberculosis patients (N = 532)

| Variable | Total n (%) |
| --- | --- |
| Age (years) | |
| 0–18 | 39 (7.4) |
| 19–30 | 139 (26.1) |
| 31–45 | 171 (32.1) |
| >45 | 183 (34.3) |
| Gender | |
| Male | 239 (44.9) |
| Female | 293 (55.1) |
| Residence | |
| Urban | 271 (51.0) |
| Rural | 261 (49.0) |
| Comorbidities | |
| Diabetes mellitus | 74 (14.0) |
| HIV infection | 26 (5.0) |
| Other (CKD, malignancy, etc.) | 26 (5.0) |
| Underweight (BMI <18.5 kg/m²) | 335 (63.0) |
| Previous history of TB | 46 (8.6) |
| Family history of TB | 63 (11.8) |
| Site of EPTB | |
| Lymph node and abscesses | 300 (56.4) |
| Pleural | 111 (21.0) |
| Abdominal | 39 (7.3) |
| CNS | 20 (3.7) |
| Genitourinary | 20 (3.7) |
| Other and disseminated | 41 (7.7) |
| Presenting symptoms | |
| >2 symptoms | 425 (80.0) |
| Fever | 260 (48.8) |
| Weight loss | 242 (45.4) |
| Fatigue, anorexia | 239 (44.9) |
| Night sweats | 145 (28.0) |
| Local swelling/pain | 143 (26.8) |
| Cough/respiratory symptoms | 103 (19.4) |
| Neurological deficits | 26 (4.8) |
| Ascites/abdominal pain | 31 (5.8) |
| Laboratory findings | |
| Elevated ESR (>50 mm/hr) | 292 (54.8) |
| Anaemia (Hb <10 g/dL) | 228 (42.8) |
| Elevated CRP (>10 mg/L) | 282 (53.0) |
| Outcomes | |
| Rifampicin-resistant | 36 (6.7) |
| Slow responders | 113 (21.2) |
