# Supplementary material for: Clinical and laboratory determinants of treatment response and rifampicin resistance among Xpert MTB/RIF-confirmed extrapulmonary tuberculosis patients: A prospective study from Central India
Source: PLoS One. 2026 Nov 2;21(11):e0361115. doi: 10.1371/journal.pone.0361115 (PMC13726920; doi:10.1371/journal.pone.0361115)
Supplement: Supplementary file 2 [file pone.0361115.s005.docx]

Supplementary Table S2. Comparison of baseline characteristics between rifampicin-resistant and slow responders extrapulmonary tuberculosis Patients.

| Characteristic | Rifampicin-Resistant (N = 36) n (%) | Slow Responders (N = 113) n (%) |
| --- | --- | --- |
| Age <30 years | 20 (55.5) | 30 (26.1) |
| Male sex | 24 (66.6) | 47 (41.6) |
| Rural residence | 21 (58.3) | 83 (73.4) |
| HIV Co-infection | 5 (13.8) | 17 (15) |
| Diabetes Mellitus | 8 (22.2) | 35 (31) |
| Previous TB history | 12 (33.3) | 14 (12.3) |
| Family History of TB | 9 (25) | 18 (16) |
| Underweight (BMI <18.5 kg/m²) | 25 (69.4) | 54 (47.7) |
| Lymph node/Abscess TB | 15 (41.6) | 35 (31) |
| Pleural TB | 4 (11.1) | 28 (25) |
| CNS TB | 3 (8.3) | 8 (7.1) |
| Abdominal TB | 3 (8.3) | 18 (16) |
| Anaemia (Hb <10 g/dL) | 16 (44.4) | 57 (50.4) |
| ESR >50 mm/hr | 30 (83.3) | 72 (63.7) |
| CRP >10 mg/L | 27 (75) | 77 (68) |
